# When BERT meets Bilbo: a learning curve analysis of pretrained language model on disease classification

**DOI:** 10.1186/s12911-022-01829-2

**Published:** 2022-04-05

**Authors:** Xuedong Li, Walter Yuan, Dezhong Peng, Qiaozhu Mei, Yue Wang

**Affiliations:** 1 College of Computer Science, Sichuan University, Chengdu, China; 2 MobLab Inc., Pasadena, CA USA; 3 School of Information, University of Michigan, Ann Arbor, MI USA; 4 School of Information and Library Science, University of North Carolina at Chapel Hill, Chapel Hill, NC USA

**Keywords:** Learning curve, Bidirectional encoder representations from transformers, Disease classification

## Abstract

**Background:**

Natural language processing (NLP) tasks in the health domain often deal with limited amount of labeled data due to high annotation costs and naturally rare observations. To compensate for the lack of training data, health NLP researchers often have to leverage knowledge and resources external to a task at hand. Recently, pretrained large-scale language models such as the Bidirectional Encoder Representations from Transformers (BERT) have been proven to be a powerful way of learning rich linguistic knowledge from massive unlabeled text and transferring that knowledge to downstream tasks. However, previous downstream tasks often used training data at such a large scale that is unlikely to obtain in the health domain. In this work, we aim to study whether BERT can still benefit downstream tasks when training data are relatively small in the context of health NLP.

**Method:**

We conducted a learning curve analysis to study the behavior of BERT and baseline models as training data size increases. We observed the classification performance of these models on two disease diagnosis data sets, where some diseases are naturally rare and have very limited observations (fewer than 2 out of 10,000). The baselines included commonly used text classification models such as sparse and dense bag-of-words models, long short-term memory networks, and their variants that leveraged external knowledge. To obtain learning curves, we incremented the amount of training examples per disease from small to large, and measured the classification performance in macro-averaged $F_{1}$ score.

**Results:**

On the task of classifying all diseases, the learning curves of BERT were consistently above all baselines, significantly outperforming them across the spectrum of training data sizes. But under extreme situations where only one or two training documents per disease were available, BERT was outperformed by linear classifiers with carefully engineered bag-of-words features.

**Conclusion:**

As long as the amount of training documents is not extremely few, fine-tuning a pretrained BERT model is a highly effective approach to health NLP tasks like disease classification. However, in extreme cases where each class has only one or two training documents and no more will be available, simple linear models using bag-of-words features shall be considered.

## Background

Machine learning has become the predominant approach to health natural language processing (NLP) in recent years. To achieve high performance, machine learning models often need to be trained on a substantial amount of labeled data. Deep learning models, while capable of achieving even higher performance, may need more training data to train a large number of internal parameters.

Unlike machine learning tasks in the general domain where training data are abundant, health NLP data are mostly small, as creating such data at scale can be prohibitively expensive and even infeasible.^1^ For instance, labeling social media posts can be crowdsourced at a very low cost through Amazon Web Services [1], while annotating clinical notes requires special medical training and long hours [2]. On the task of rare disease identification, the amount of labeled documents is further bounded by the size of population, since rare diseases appear very infrequently (a rare disease affects fewer than 1 in 1500 people in the U.S. [3] or 1 in 2000 in Europe [4]). As a result, health NLP researchers have been proposing a variety of methods to compensate for the lack of training data [5]. These include leveraging expert knowledge and medical ontologies [6–8], transferring statistical knowledge learned from related tasks [9], simultaneously learning from multiple tasks [10], using weak/distant supervision signals [11, 12], selectively asking experts for label [13].

Recently, Bidirectional Encoder Representations from Transformers (BERT) model has been increasingly adopted by the NLP research community as it celebrates superior performance in a wide range of NLP tasks [14]. BERT learns contextual representation of words using information from both sides of a word, effectively capturing syntactic and semantic knowledge that can benefit many NLP tasks. A pretrained BERT model can be tailored to a specific NLP task by using the task-specific data to further train the model, a procedure known as ``fine-tuning''. In this way, the new task can build on top of the pretrained knowledge in BERT to achieve superior generalization performance. However, previous works all use very large data sets for fine-tuning, which are often on the order of hundreds of thousands and even millions of examples [15, 16]. In general, however, it is impractical to collect training data at such a large scale in the health domain, for reasons discussed above.

Given the high potential of BERT and the often small data in health NLP, it is natural to ask the following question: *can we fine-tune BERT on small health NLP data and still achieve superior performance?* On the one hand, BERT may hold the promise as it has been shown to perform well in many NLP tasks thanks to the unsupervised pretraining. On the other hand, BERT is itself a large complex model with a massive number of parameters, so to achieve high performance it may need a good amount of labeled data for fine-tuning.

**Fig. 1:**
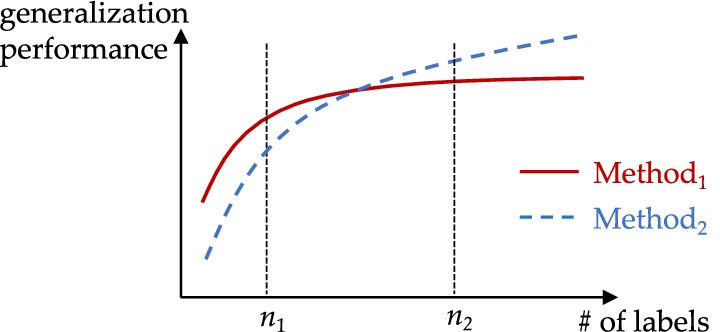
Learning curves can inform NLP method selection given labeling budget. If the labeling budget is $n_{1}$, then $Method_{1}$ is preferred. If the labeling budget increases to $n_{2}$, then $Method_{2}$ is preferred

In this paper, we answer the above question by conducting learning curve analyses of BERT and other models on a disease diagnosis task. As conceptually shown in Fig. 1, a learning curve can be viewed as a ''return-on-investment'' curve, where the ''investment'' is labeled data, and the ''return'' is a model's generalization performance on test data. Learning curves allow us to compare the performance of different models given different labeling budgets. They can also show which model will improve faster if we invest more labels. Such a comparison is especially relevant when the labeling cost is high, as in health NLP task scenarios.

The learning curve analysis reveals a series of interesting and informative findings, as summarized below:

- BERT is able to achieve superior performance even when fine-tuned on a handful of (but more than one) labeled documents per class.
- BERT's prior knowledge can effectively compensate for the lack of training data in most cases, but simple linear models are still worth considering when the amount of training data is extremely limited and not expected to increase any time soon. In the extreme case where each class has only one or two labeled documents, BERT could be outperformed by models using carefully engineered sparse bag-of-words features.
- When more labeled documents start to become available, BERT demonstrates fast rate of performance gain, which allows it to quickly outperform other models by a significant margin. It shows that BERT's prelearned representation enables it to extract the most rich information from each training example. In other words, if we modestly increase the labeling budget, BERT will likely show a very high return.

### Prior work

#### BERT in health domain

Lee et al. obtained BioBERT by taking Google's pretrained BERT model and continuing the pretraining tasks (masked language modeling and next sentence prediction) on large-scale biomedical literature [17]. The use of domain-specific texts enabled BioBERT to outperform BERT on certain biomedical NLP tasks. Alsentzer et al. [18] further added clinical texts to continue the pretraining on the basis of BioBERT to get Clinical BERT. A closely related line of work was conducted by Peng et al., where BERT is fine-tuned on biomedical and clinical texts, and then applied to ten benchmarking tasks, including sentence similarity measurement, named entity recognition, relation extraction, document classification, and logical inference [19]. All the above works demonstrate the value of domain-specific pretraining when applying BERT on health domain tasks. BERT has also been applied to non-English health NLP tasks. Pretrained Chinese BERT models have been fine-tuned and applied on NLP tasks such as disease classification, [20], named entity recognition [21], and a host of other tasks [22].

This paper studies BERT from another significant perspective, i.e., its generalization performance when fine-tuned on small training data. To the best of our knowledge, there has been no previous work that studies the performance of BERT when the size of training data starts from very small. Instead, researchers often use learning curves to demonstrate the enormous learning capacity of deep learning models when training data size scales up exponentially [23].

#### Disease classification

Stanfill et al. conducted a systematic literature review of clinical coding and classification systems [24]. Recent works on disease classification studied various application scenarios, including smoking status identification [25], obesity prediction [26, 27], online patient forum moderation [28], cancer patient sentiment classification [29], vaccine adverse events detection [30], etc. These works above are all based on English texts. Zhong et al. [31] applied nearest neighbor classifier to identify the disease category based on patient disease description in Chinese. In this study, we predict the presence of a disease in documents written in Chinese. Although the texts are written by patients and health insurance professionals, applying NLP on these texts shares similar challenges as clinical NLP [32, 33], where the texts are written by physicians.

#### Incorporating existing knowledge

External knowledge has significant impact on machine learning performance. Besides pretraining model parameters using large unlabeled corpus, incorporating knowledge from ontologies (a.k.a. knowledge graphs or KGs) has also received attention. Garla et al. [34] utilized the relationship between medical concepts in KG to improve feature selection. Yao et al. used UMLS entity embeddings in convolutional neural networks [27]. Li et al. used KG to derive additional knowledge features in rare disease classification [35]. Choi et al. [36] developed a graph-based attention model to represent words using node vectors learned from the ontology. Some studies [37, 38] suggest that incorporating KG into BERT also can bring some benefits.

## Method

### Data description and problem formulation

We use two Chinese patient disease classification corpora. The first corpus, HaoDaiFu, contains 51,374 patient records categorized into 805 diseases. Each document contains the symptom description submitted by a patient to Haodf.com, the largest Chinese online platform that connects patients to doctors. These patients have been previously diagnosed by a clinician, and now come to the platform for further consultation. The second corpus, ChinaRe, contains 86,663 patient records categorized into 44 disease categories. Each document contains the symptom description of a patient written by a health insurance professional in ChinaRe, which is one of the largest reinsurance groups in China. The diagnoses were determined by a clinician and sent to the insurance company. In both corpora, each document corresponds to a unique patient and only has one disease label. Table 1 summarizes basic statistics of the two corpora. Jieba package was used for Chinese word segmentation [39].

**Table 1:** Corpora statistics

	HaoDaiFu	ChinaRe
# of documents	51,374	86,663
# of diseases	805	44
# of rare diseases	89	5
Vocabulary size	59,879	41,087
Average # of words/doc	27	30

#### Problem formulation

The task of patient diagnosis can be formulated as a text classification problem: to assign a disease label given the narrative description of a patient's symptoms. Accurate disease diagnosis is an important task towards computer-assisted patient triage and risk stratification. We aim to study the performance of different classification models (especially comparing BERT to other models) when provided an increasing amount of training data.

### Compared algorithms

In this section, we describe classification models we include in comparison. We include text classification models that use one-hot word representations, distributed word representations, and contextual word representations. Since our main goal here is to study the behavior of classifiers when the training data size increases from small to large, we do not consider classification techniques intended for small data sizes only, e.g. one-shot learning or few-shot learning classifiers.

### Classifiers using one-hot word representations

We first consider the most common baseline of text classification—a linear classifier using bag-of-words features (and its variants). Although simple, such a model offers two advantages in handling small training data. First, a regularized, sparse linear classifier does not overfit as easily as complex models, therefore delivering stable performance. Second, the simple model allows relatively straightforward ways of incorporating prior knowledge into its feature representation.

#### BOW

This is a support vector machine classifier using TFIDF-weighted bag-of-words (BOW) features and linear kernel, trained with $L_{2}$ regularization.

#### BOW_EXP

This model enhances the feature representation of BOW with feature selection and synonym *exp*ansion techniques. The basic idea is to emphasize class-indicative features in a document if that document contains such a feature or its synonyms. It takes the following steps:

- A feature selection algorithm is used to rank the relevance of each unigram feature in the classification task.
- Each unigram feature $w$ is associated with a class $c$ if $c$ has the largest $p\left( {c{|}w} \right)$ in training data. For each class, we select $k$ highest ranking features according to the feature selection metric. The union of all selected features are denoted as $F$.
- For each word $u$ in a document $d$, we compute its vector similarity to the vector of each $w \in F$ in a word embedding space. If cosine similarity $\cos \left( {\vec{u},\vec{w} \ge {\text{t}}} \right)$, we increment the count of $w \in d$ by 1 before computing the TFIDF transformation. The step conceptually adds a new word $w$ into $d$.

The above algorithm is a hybrid of feature selection and feature expansion [40]. Instead of discarding unselected features (which may still be useful), it increases the weights of selected features in each document. The method is inspired by the distributional prototype features proposed by [41] and later applied in clinical NLP [42].

#### BOW_EXP_KG

This model refines BOW_EXP by using knowledge graph (KG)-enhanced word vectors. A knowledge graph can be viewed as a semantic network, where entities (words and phrases) are nodes and relations between concepts are edges. We employ the LINE network embedding algorithm to learn low-dimensional word vectors that preserve knowledge in the semantic network [43].

### Classifiers using distributed word representations

We consider another group of text classification models that represent words as distributed semantic vectors [44]. These word vectors can be learned from scratch using the data of current task, or initialized with word vectors learned on related tasks to transfer semantic knowledge. Here we consider two representative models using distributed word vectors: the continuous bag-of-words model and long short-term memory networks.

#### CBOW

This is a linear-kernel support vector machine classifier that represents a document as the average of its words' vectors. It is also known as continuous bag-of-words (CBOW) [45], as conventional bag-of-words representation can be viewed as an average of one-hot word vectors. The word vectors are the same as in BOW_EXP and fixed in the training process.

#### CBOW_KG

This model refines CBOW by using KG-enhanced word vectors as used in BOW_EXP_KG.

#### LSTM

This classifier uses unidirectional long short-term memory networks (LSTM) to process the document as a word sequence. The model's word embedding layer is initialized with the same word vector as in BOW_EXP and fine-tuned in the training process.

#### LSTM_KG

This model refines LSTM by initializing the word embedding layer with KG-enhanced word vectors as used in BOW_EXP_KG and CBOW_KG. The word embedding layer is fine-tuned in the training process.

### Classifier using contextual word representations

Exemplified by BERT (Bidirectional Encoder Representations from Transformers [46]), contextual word representations encode each word using not only the distributed vector of the word itself, but also distributed vectors of surrounding words that have semantic dependencies with the word [47]. BERT extensively uses multi-head attention mechanism to represent each word by "paying attention to'' all other words in the same context (sentence or document). Instead of processing tokens sequentially as in LSTM, BERT's multi-head attention can process all tokens in parallel. This mitigates the gradient vanishing problem when capturing long-range dependencies between words. As a result, BERT can efficiently model the dependencies between labels and words as well as among words themselves.

#### BERT

We configure a Chinese BERT-base model released by Google^2^ to perform multiclass classification tasks. Since the primary goal of this study is to compare BERT with other non-BERT classification models on small training data, it suffices to use a BERT model pretrained on general domain texts. We leave the study that compares BERT models fine-tuned on Chinese clinical texts [21, 22] for future work.

This sentence has two reference citations [1, 2].

More text of an additional paragraph, with a figure reference (Fig. 1) and a figure inside a Word text box below. Figures need to be placed as close to the corresponding text as possible and not extend beyond one page.

### Implementation details

The support vector machine classifier (SVM) was implemented using Python scikit-learn package. To determine the best regularization strength $C$ for SVM models, we performed grid search over {0.001, 0.01, 0.1, 1, 10, 100} on a development set. We set $C = 1$ as it consistently delivered the best result (performance metric discussed below).

We explored various feature selection algorithms used in BOW_EXP and BOW_EXP_KG. These include chi-square ${\upchi }^{2}$, information gain, and bi-normal separation [48] in our pilot study. We selected the ${\upchi }^{2}$ method as it delivers the best performance on development set. We select $k = 2$ features for each class.

In BOW_EXP and BOW_EXP_KG, the threshold of cosine similarity was set to $t = 0.9$ after searching over {0.7, 0.8, 0.9} on development set.

We used 256-dimensional word vectors pretrained on a large-scale Chinese text corpus [49] in BOW_EXP_KG and CBOW_KG.

To learn KG-enhanced word vectors, we derive a semantic network from a general Chinese knowledge graph, CN-DBpedia [50]. It contains 16.8 million entities and 223 million relations and is publicly available.^3^

We used the LINE network embedding algorithm to fine-tune word vectors using the massive semantic network above. It was configured to learn from secondary-order proximity. We performed grid search for LINE's hyperparameters on a development set. These include (the best setting is underlined): negative edge sampling rate {5, 10, 50, 100}, batch size {128, 256, 512, 1024, 2048}, and number of batches {50 K, 100 K, 150 K, 200 K, 250 K, 300 K}.

We used tensorflow/keras to implement deep sequence learning models, including LSTM, LSTM_KG, and BERT. For LSTM models, we used the recommended Adam optimizer and default learning rate ($10^{ - 3}$). We set the number of training epochs such that the loss on validation set stops decreasing. For BERT, we also used the recommended Adam optimizer and default learning rate decaying schedule. The number of training epochs was set to 40 using the same procedure as LSTM models. A document is padded (truncated) if it is shorter (longer) than the maximum sequence length supported by BERT-base (512 words).

### Evaluation methodology

#### Performance metric

Viewing the classification of each individual disease (class) as a binary classification problem, results can be divided into True Positive (TP), True Negative (TN), False Positive (FP), and False Negative (FN). *Recall* measures the percentage of TPs among all documents that truly mention that disease; *precision* measures the percentage of TPs among all documents predicted to mention that disease. $F_{1}$ score is the harmonic mean of precision and recall, a metric that balances the two [51]. To measure the classification performance of a set of diseases, we use macro-averaged $F_{1}$. Formally, the metrics are calculated as follow

1 $$\begin{array}{*{20}c} {F_{1} = \frac{2 \times recall \times precision}{{recall + precision}} = \frac{2 \times TP}{{2 \times TP + FP + FN}},} \\ \end{array}$$

2 $$\begin{array}{*{20}c} {macro - averagedF_{1} = \frac{1}{\left| D \right|}\mathop \sum \limits_{i = 1}^{\left| D \right|} F_{1,i} ,} \\ \end{array}$$

where $D$ is the set of diseases (classes), and $F_{1,i}$ is the $F_{1}$ score of the *i-*th disease.

#### Train-test split

To reduce the variance of results due to a random train-test split, we average the results of 10 runs. In each run, we randomly split the corpus into 80% for training and 20% for test. To avoid the case where some classes do not appear in training or test set, the random split is applied on a per-class basis.

#### Learning curve

The results of evaluation metrics we mentioned above are displayed in plots of learning curves. Learning curves represent the generalization performance of the models produced by a learning algorithm, as a function of the size of the training set. In a plot of learning curve, *x*-axis represents the size of training set, *y*-axis represents the performance of model under an evaluation metric. In our study, we sample training sets from total training examples in fixed proportions: [10%, 20%, 30%, 40%, 50%, 60%, 70%, 80%, 90%, 100%].

We use Area Under Learning Curve (ALC) to summarize the learning progress of each model. The ALC metric is useful in comparing different learning algorithms especially when labeling budget is limited, as in the active learning setting [52]. A higher ALC means an overall higher performance across different training data sizes.

### Experimental evaluation

**Fig. 2:**
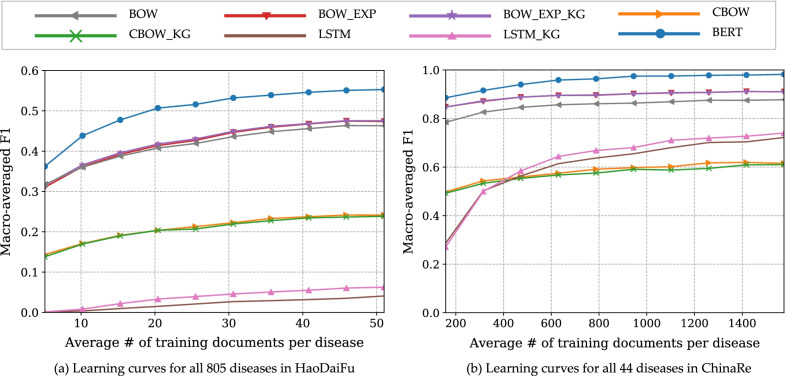
Learning curves of compared algorithms averaged across all diseases in the two corpora

The learning curves of different algorithms on HaoDaiFu and ChinaRe corpora are in Fig. 2, with their corresponding ALC metrics reported in Table 2. On both corpora, BOW_EXP, BOW_EXP_KG, and BERT significantly outperformed the BOW baseline, and BERT significantly outperformed the BOW_EXP_KG method.

**Table 2:** Area under learning curve (ALC) for different methods aggregated over all diseases

Method	HaoDaiFu (all 805 diseases)	ChinaRe (all 44 diseases)
BOW	0.4158	0.8534
BOW_EXP	0.4266^a^	0.8934^a^
BOW_EXP_KG	0.4254^a^	0.8940^a^
CBOW	0.2097	0.5817
CBOW_KG	0.2064	0.5714
LSTM	0.2013	0.6064
LSTM_KG	0.0377	0.6243
BERT	0.5020^ab^	0.9551^ab^

Figure 2 plots the learning curves

^a^Result significantly higher than BOW

^b^Result significantly higher than BOW_EXP_KG. (Fisher's randomization test, significance level ${\upalpha } = 0.05$)

**Fig. 3:**
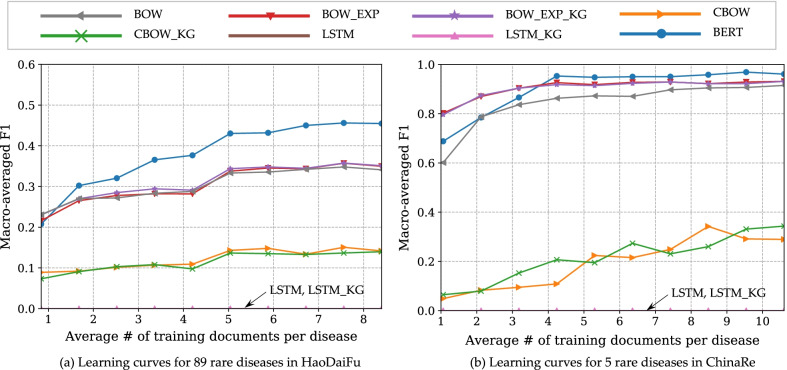
Learning curves of compared algorithms averaged across very rare diseases (prevalence $\le$ 0.02%) in the two corpora

To further study the behavior of different algorithms when training data are extremely few, we plot the learning curves on statistically rare diseases that account for no more than 0.02% (2 in 10,000) of records in each corpus. There are 89 such diseases in HaoDaiFu and 5 in ChinaRe. In both cases, these extremely rare diseases have on average about 10 training documents. This translates to *one training document per disease* at 10% training data rate, representing the cases of extreme data scarcity. The corresponding learning curves are in Fig. 3, ALC metrics reported in Table 3. On HaoDaiFu, BOW_EXP, BOW_EXP_KG, and BERT significantly outperformed the BOW baseline on all diseases, and BERT significantly outperformed the BOW_EXP_KG method on extremely rare diseases. Since the number of rare diseases in ChinaRe is too few, the above performance comparisons did not show significant differences.

**Table 3:** Area under learning curve (ALC) for different methods aggregated over extremely rare ($prevalence \le 0.02$)

Method	HaoDaiFu (89 rare diseases)	ChinaRe (5 rare diseases)
BOW	0.3044	0.8454
BOW_EXP	0.3056^a^	0.9058
BOW_EXP_KG	0.3115^a^	0.9034
CBOW	0.1215	0.1945
CBOW_KG	0.1153	0.2136
LSTM	0	0
LSTM_KG	0	0
BERT	0. 3795^ab^	0.9028

Figure 3 plots the learning curves

^a^Result significantly higher than BOW

^b^Result significantly higher than BOW_EXP_KG. (Fisher's randomization test, significance level $\alpha = 0.05$)

Note that the classification performance on HaoDaiFu is overall lower than that on ChinaRe. In general, multi-class classification problem is difficult with a large number of classes. Here the HaoDaiFu corpus contains one order of magnitude more classes than ChinaRe (see Table 1), bringing substantial challenge to all methods.

## Results

Learning curves of different algorithms on HaoDaiFu and ChinaRe corpora are in Fig. 2, with their corresponding ALC metrics reported in Table 2. On both corpora, BOW_EXP, BOW_EXP_KG, and BERT significantly outperformed the BOW baseline, and BERT significantly outperformed the BOW_EXP_KG method.

To further study the behavior of different algorithms when training data are extremely few, we plot the learning curves on statistically rare diseases that account for no more than 0.02% (2 in 10,000) of records in each corpus. There are 89 such diseases in HaoDaiFu and 5 in ChinaRe. In both cases, these extremely rare diseases have on average about 10 training documents. This translates to *one training document per disease* at 10% training data rate, representing the cases of extreme data scarcity. The corresponding learning curves are in Fig. 3, ALC metrics reported in Table 3. On HaoDaiFu, BOW_EXP, BOW_EXP_KG, and BERT significantly outperformed the BOW baseline on all diseases, and BERT significantly outperformed the BOW_EXP_KG method on extremely rare diseases. Since the number of rare diseases in ChinaRe is too few, the above performance comparisons did not show significant differences.

Note that the classification performance on HaoDaiFu is overall lower than that on ChinaRe. In general, multi-class classification problem is difficult with a large number of classes. Here the HaoDaiFu corpus contains one order of magnitude more classes than ChinaRe (*c.f.* Table 1), bringing substantial challenge to all methods.

## Discussion

The area under BERT's learning curve is the largest when aggregated across all diseases when aggregated across all diseases. With a fraction of all training data (30% on Haodaifu, and 40% on ChinaRe), BERT is able to outperform all other approaches trained on 100% training data. These results show that BERT not only can deliver the best performance but also requires less data for training compared to other methods. The outstanding performance partly comes from Transformer's multi-head attention mechanism, which allows BERT to learn long-distance dependency much more efficiently than previous deep sequence models. It is also partly due to the unique pretraining objective, which can incorporate the sequence information of text in two directions efficiently.

BOW gives a decent baseline performance. Its variants, BOW_EXP and BOW_EXP_KG, give consistent performance improvements. Supervised feature selection and synonym expansion effectively improve the feature representation of BOW baseline. BOW_EXP_KG only gives slightly higher performance than BOW_EXP. This indicates that semantic relation information in a knowledge graph is already largely captured by pretrained word vectors.

CBOW performs worse than BOW. Similar result was observed in [53]. Indeed, linear SVM aims to find hyperplanes in the feature space to separate classes. It is easier to achieve linear separation in the high dimensional sparse feature space (BOW) than in the low dimensional dense feature space (CBOW).

The performance of LSTM on Haodaifu is extremely low, but is not that bad on ChinaRe, and goes up sharply when training data increases from 10 to 40%. This huge difference reflects the model's requirement for a large quantity of training data. On average, there are 51 training documents per disease in Haodaifu, while 1575 training documents per disease in ChinaRe. Because of the vanishing gradient problem, training LSTM models becomes extremely difficult when training data size is small and documents are relatively long. Adding prior knowledge through word embedding (LSTM_KG) has only limited benefit.

On the extremely rare diseases (when there is only 1 training document per disease), BERT is outperformed by BOW_EXP and BOW_EXP_KG. This happened on both Haodaifu and ChinaRe. The result shows that in situations where training data is extremely scarce, the traditional non-deep model with an appropriate feature construction strategy is able to compete with the current state-of-the-art deep models.

On rare diseases, the catastrophically low performance of LSTM models is not unexpected given its poor performance on all diseases. Again, the result suggests that a large amount of training data is needed to train LSTM models, even though its word embedding layer has been pretrained.

### Implication

Medical domain has accumulated a wealth of knowledge bases, in the form of standardized terminologies, research publications, clinical practice guidelines, and consumer-facing information portals. While these forms of knowledge can be easily used by humans, they cannot be directly used by machine learning models. This is because the internal representation of knowledge in machine learning models is fundamentally different from that of human knowledge. The primary way of transferring knowledge into these models is through well-formulated prediction tasks expressed in the form of labeled examples. However, labeling cost is high in the medical domain, necessitating machine learning models to leverage medical domain knowledge. Over the years, researchers have been proposing various approaches for instilling external knowledge into machine learning models, including carefully designed features, model architectures, auxiliary learning objectives [9, 10], weak labels and distant supervision obtained from medical knowledge bases [8, 11, 12], pretrained model parameters [17, 18, 22], and combinations of these approaches.

Our study here shows that pretrained BERT models (and the broader family of pretrained deep Transformers) may offer an effective way of leveraging external knowledge learned from large-scale unlabeled data towards specific NLP tasks. Even a BERT model pretrained on general corpus is able to effectively help NLP tasks in the health domain. On the one hand, this is good news to the health NLP research community, as it can potentially free researchers from feature engineering when the training data is small and the labeling cost is high. Instead, the model can be continuously improved by pretraining on domain-specific and task-specific corpora [17, 18, 54]. On the other hand, these black-box models are difficult to interpret, therefore more research is needed to understand their vulnerabilities especially within the medical context, such as potential biases in learned representations [55].

## Conclusion

In this paper, we study whether BERT is still effective when it is fine-tuned with small training data. To answer this question, we conducted a learning curve analysis of BERT and other baseline models in text-based disease classification tasks. The analysis showed that BERT remains the highest performing model even when each class has only a handful of training documents, and its performance improves the fastest when given more training documents. Simple linear classifiers using specially engineered bag-of-words features delivers stable and competitive performance, and it outperformed BERT when training documents are extremely few (one or two per class). Overall, the study shows that even though BERT is a massively complex model, it only takes very small (but not extremely small) training data to fine-tune a pretrained BERT model to outperform baseline approaches using the same data.

## Data Availability

The ChinaRe dataset in this paper is proprietary. The HaoDaiFu dataset is publicly available at https://github.com/bruceli518/HaoDaiFu.

## Abbreviations

NLP: Natural language processing
BERT: Bidirectional encoder representations from transformers
KG: Knowledge graph
UMLS: Unified medical language system
BOW: Bag-of-words
CBOW: Continuous bag-of-words
LSTM: Long short-term memory

## Footnotes

1 In *The Lord of the Rings*, ''Bert'' is a giant stone troll. In terms of size, health NLP data are the ''Hobbits'' (represented by Bilbo Baggins) among machine learning datasets.

2 https://github.com/google-research/bert.

3 http://kw.fudan.edu.cn/cndbpedia/download/.

## References

[1] Amazon Web Services. Amazon SageMaker Ground Truth pricing. https://aws.amazon.com/sagemaker/groundtrut. Accessed July 2020.

[2] Stubbs A, Uzuner Ö (2015). Annotating longitudinal clinical narratives for de-identification: the 2014 i2b2/UTHealth corpus. J Biomed Inform.

[3] United States Department of Health and Human Services. National Organization for Rare Disorders (NORD); Last Updated June 23, 2020. https://www.nidcd.nih.gov/directory/national-organization-rare-disorders-nord. Accessed 23 June 2020.

[4] European Commission. Rare Diseases. https://ec.europa.eu/health/non-communicable-diseases/steering-group/rare-diseases_en. Accessed 16 July 2020.

[5] Spasic I, Nenadic G (2020). Clinical text data in machine learning: systematic review. JMIR Med Inform.

[6] Wilcox AB, Hripcsak G (2003). The role of domain knowledge in automating medical text report classification. J Am Med Inform Assoc.

[7] Demner-Fushman D, Mork JG, Shooshan SE, Aronson AR (2010). UMLS content views appropriate for NLP processing of the biomedical literature vs. clinical text. J Biomed Inform.

[8] Dissanayake PI, Colicchio TK, Cimino JJ (2020). Using clinical reasoning ontologies to make smarter clinical decision support systems: a systematic review and data synthesis. J Am Med Inform Assoc.

[9] Zhang E, Thurier Q, Boyle L (2018). Improving clinical named-entity recognition with transfer learning. Stud Health Technol Inform.

[10] Crichton G, Pyysalo S, Chiu B, Korhonen A (2017). A neural network multi-task learning approach to biomedical named entity recognition. BMC Bioinform.

[11] Wang Y, Sohn S, Liu S, Shen F, Wang L, Atkinson EJ (2019). A clinical text classification paradigm using weak supervision and deep representation. BMC Med Inform Decis Mak.

[12] Pattisapu N, Anand V, Patil S, Palshikar G, Varma V (2020). Distant supervision for medical concept normalization. J Biomed Inform.

[13] Figueroa RL, Zeng-Treitler Q, Ngo LH, Goryachev S, Wiechmann EP (2012). Active learning for clinical text classification: is it better than random sampling?. J Am Med Inform Assoc.

[14] Devlin J, Chang MW, Lee K, Toutanova K. Bert: pre-training of deep bidirectional transformers for language understanding. arXiv:1810.04805. 2018.

[15] Adhikari A, Ram A, Tang R, Lin J. Docbert: Bert for document classification. arXiv:1904.08398. 2019.

[16] Wang A, Singh A, Michael J, Hill F, Levy O, Bowman SR. Glue: a multi-task benchmark and analysis platform for natural language understanding. arXiv:1804.07461. 2018.

[17] Lee J, Yoon W, Kim S, Kim D, Kim S, So CH (2020). BioBERT: a pre-trained biomedical language representation model for biomedical text mining. Bioinformatics.

[18] Alsentzer E, Murphy JR, Boag W, Weng WH, Jin D, Naumann T, et al. Publicly available clinical BERT embeddings. arXiv:1904.03323. 2019.

[19] Peng Y, Yan S, Lu Z. Transfer learning in biomedical natural language processing: an evaluation of bert and elmo on ten benchmarking datasets. arXiv:1906.05474. 2019.

[20] Yao L, Jin Z, Mao C, Zhang Y, Luo Y (2019). Traditional Chinese medicine clinical records classification with BERT and domain specific corpora. J Am Med Inform Assoc.

[21] Li X, Zhang H, Zhou XH (2020). Chinese clinical named entity recognition with variant neural structures based on BERT methods. J Biomed Inform.

[22] Zhang N, Jia Q, Yin K, Dong L, Gao F, Hua N. Conceptualized representation learning for chinese biomedical text mining. arXiv:2008.10813. 2020.

[23] Hestness J, Narang S, Ardalani N, Diamos G, Jun H, Kianinejad H, et al. Deep learning scaling is predictable, empirically. arXiv:1712.00409. 2017.

[24] Stanfill MH, Williams M, Fenton SH, Jenders RA, Hersh WR (2010). A systematic literature review of automated clinical coding and classification systems. J Am Med Inform Assoc.

[25] Uzuner Ö, Goldstein I, Luo Y, Kohane I (2008). Identifying patient smoking status from medical discharge records. J Am Med Inform Assoc.

[26] Uzuner Ö (2009). Recognizing obesity and comorbidities in sparse data. J Am Med Inform Assoc.

[27] Yao L, Mao C, Luo Y (2019). Clinical text classification with rule-based features and knowledge-guided convolutional neural networks. BMC Med Inform Decis Mak.

[28] Huh J, Yetisgen-Yildiz M, Pratt W (2013). Text classification for assisting moderators in online health communities. J Biomed Inform.

[29] Edara DC, Vanukuri LP, Sistla V, Kolli VKK (2019). Sentiment analysis and text categorization of cancer medical records with LSTM. J Ambient Intell Humaniz Comput.

[30] Botsis T, Nguyen MD, Woo EJ, Markatou M, Ball R (2011). Text mining for the vaccine adverse event reporting system: medical text classification using informative feature selection. J Am Med Inform Assoc.

[31] Zhong J, Yi X, Xuan D, Xie Y, Perner P (2018). Categorization of patient diseases for chinese electronic health record analysis: a case study. Industrial conference on data mining.

[32] Friedman C, Kra P, Rzhetsky A (2002). Two biomedical sublanguages: a description based on the theories of Zellig Harris. J Biomed Inform.

[33] Wu S, Roberts K, Datta S, Du J, Ji Z, Si Y (2020). Deep learning in clinical natural language processing: a methodical review. J Am Med Inform Assoc.

[34] Garla VN, Brandt C (2012). Ontology-guided feature engineering for clinical text classification. J Biomed Inform.

[35] Li X, Wang Y, Wang D, Yuan W, Peng D, Mei Q (2019). Improving rare disease classification using imperfect knowledge graph. BMC Med Inform Decis Mak.

[36] Choi E, Bahadori MT, Song L, Stewart WF, Sun J. GRAM: graph-based attention model for healthcare representation learning. In: Proceedings of the 23rd ACM SIGKDD international conference on knowledge discovery and data mining; 2017. p. 787–95. 10.1145/3097983.3098126 PMC7954122 33717639

[37] Zhang Z, Han X, Liu Z, Jiang X, Sun M, Liu Q. ERNIE: enhanced language representation with informative entities. arXiv:1905.07129. 2019.

[38] Liu W, Zhou P, Zhao Z, Wang Z, Ju Q, Deng H, et al. K-bert: enabling language representation with knowledge graph. arXiv:1909.07606. 2019.

[39] Jieba Chinese text segmentation. https://github.com/fxsjy/jieba. Accessed 26 Mar 2019.

[40] Gabrilovich E, Markovitch S (2005). Feature generation for text categorization using world knowledge. IJCAI.

[41] Guo J, Che W, Wang H, Liu T. Revisiting embedding features for simple semi-supervised learning. In: Proceedings of the 2014 conference on empirical methods in natural language processing (EMNLP); 2014. p. 110–20.

[42] Wu Y, Xu J, Jiang M, Zhang Y, Xu H. A study of neural word embeddings for named entity recognition in clinical text. In: AMIA annual symposium proceedings, vol. 2015. American Medical Informatics Association; 2015. p. 1326. PMC4765694 26958273

[43] Tang J, Qu M, Wang M, Zhang M, Yan J, Mei Q. Line: large-scale information network embedding. In: Proceedings of the 24th international conference on world wide web; 2015. p. 1067–77.

[44] Mikolov T, Sutskever I, Chen K, Corrado GS, Dean J (2013). Distributed representations of words and phrases and their compositionality. Adv Neural Inf Process Syst.

[45] Mikolov T, Chen K, Corrado G, Dean J. Efficient estimation of word representations in vector space. arXiv:1301.3781. 2013.

[46] Vaswani A, Shazeer N, Parmar N, Uszkoreit J, Jones L, Gomez AN (2017). Attention is all you need. Adv Neural Inf Process Syst.

[47] Smith NA. Contextual word representations: a contextual introduction. arXiv:1902.06006. 2019.

[48] Forman G (2003). An extensive empirical study of feature selection metrics for text classification. J Mach Learn Res.

[49] Su J. Pretrained Word2Vector. https://kexue.fm/archives/4304. Accessed 03 Apr 2017.

[50] Xu B, Xu Y, Liang J, Xie C, Liang B, Cui W, et al. CN-DBpedia: a never-ending Chinese knowledge extraction system. In: International conference on industrial, engineering and other applications of applied intelligent systems. Springer; 2017. p. 428–38.

[51] Wikipedia. F1 Score. https://en.wikipedia.org/wiki/F1_score. Accessed 26 Mar 2019.

[52] Guyon I, Cawley GC, Dror G, Lemaire V. Results of the active learning challenge. In: Active learning and experimental design workshop in conjunction with AISTATS 2010; 2011. p. 19–45.

[53] Lilleberg J, Zhu Y, Zhang Y. Support vector machines and word2vec for text classification with semantic features. In: 2015 IEEE 14th international conference on cognitive informatics & cognitive computing (ICCI*CC). IEEE; 2015. p. 136–40.

[54] Gururangan S, Marasovic A, Swayamdipta S, Lo K, Beltagy I, Downey D, et al. Don’t stop pretraining: adapt language models to domains and tasks. In: Proceedings of the 58th annual meeting of the association for computational linguistics. Online: Association for Computational Linguistics; 2020. p. 8342–60. https://aclanthology.org/2020.acl-main.740/.

[55] Zhang H, Lu AX, Abdalla M, et al. Hurtful words: quantifying biases in clinical contextual word embeddings. In: Proceedings of the ACM conference on health, inference, and learning. 2020. p. 110–20.

